# Using a motion capture system for spatial localization of EEG electrodes

**DOI:** 10.3389/fnins.2015.00130

**Published:** 2015-04-20

**Authors:** Pedro M. R. Reis, Matthias Lochmann

**Affiliations:** Department of Sports and Exercise Medicine, Institute of Sport Science and Sport, Friedrich-Alexander-University Erlangen-NurembergErlangen, Germany

**Keywords:** electroencephalography, methodology, EEG sensor position, sensor location, x-ray computed tomography, electrodes digitalization, IR-MOCAP

## Abstract

Electroencephalography (EEG) is often used in source analysis studies, in which the locations of cortex regions responsible for a signal are determined. For this to be possible, accurate positions of the electrodes at the scalp surface must be determined, otherwise errors in the source estimation will occur. Today, several methods for acquiring these positions exist but they are often not satisfyingly accurate or take a long time to perform. Therefore, in this paper we describe a method capable of determining the positions accurately and fast. This method uses an infrared light motion capture system (IR-MOCAP) with 8 cameras arranged around a human participant. It acquires 3D coordinates of each electrode and automatically labels them. Each electrode has a small reflector on top of it thus allowing its detection by the cameras. We tested the accuracy of the presented method by acquiring the electrodes positions on a rigid sphere model and comparing these with measurements from computer tomography (CT). The average Euclidean distance between the sphere model CT measurements and the presented method was 1.23 mm with an average standard deviation of 0.51 mm. We also tested the method with a human participant. The measurement was quickly performed and all positions were captured. These results tell that, with this method, it is possible to acquire electrode positions with minimal error and little time effort for the study participants and investigators.

## 1. Introduction

Electroencephalography (EEG) is perhaps the oldest method for inspecting brain activity. It records the cortex electric activity that is projected at scalp level. This is used for a myriad of purposes ranging from clinical diagnostics to machine control. Some of these purposes require the use of source localization techniques which attempt to localize in 3D, i.e., tomographically, the cortex sources of the activity recorded at scalp level. Source localization techniques to be accurate, depend on certain factors. Factors such as the parameters of the adopted head model, the scalp area of electric activity sampling and the positions of the electrodes (Michel et al., 2004). The accurate positioning of EEG sensors allows the sampled data to be co-registered with the person's own individual anatomy.

There is a standard method for positioning EEG electrodes. This is the 10–20 system in which the distances between electrodes are either 10 or 20% of the total front-back or right-left distance of the skull (Jasper, 1958). This system has been used over many years and its limitations are well-described (Boon, 1997; Michel et al., 2004; Jurcak et al., 2007). One of the limitations of the 10–20 system is the subjective interpretation of the sensors placement. This method also does not account for small inter electrode positioning differences or the person's individual anatomy. In addition, presently most of EEG electrodes are integrated on elastic caps with more or less determined positions that adjust to the person's head (Michel et al., 2004).

There are a few systems that allow the individual measurement of the electrodes' positions. The ELPOS system (Zebris Medical GmbH, Max-Eyth-Weg 43, D-88316 Isny, Germany) and the FastTrack system (Polhemus Inc, 40 Hercules Dr, Colchester, VT 05446, United States of America) are both used for digitizing the electrodes' positions. Even though these systems offer a nice solution, they are not without flaws. Both use a stylus with which the user touches the electrode, so that the system records its relative position. Furthermore, by touching the electrode, even slightly, the user is already changing its position and, therefore, the digitized values. The ELPOS system uses ultrasound to detect the positions and the FastTrack uses electromagnetism to track the position of the stylus. These methods are subject to the room environment changes such as electromagnetic interference or air displacement. Furthermore, these methods are very user dependent, prone to user error even after extensive experience. Engels et al. (2010) explores the factors influencing the precision of the FastTrack. In spite of these limitations, many laboratories, and manufacturers use the FastTrack system as the standard system to digitize electrodes.

Other user independent methods also exist, such as the Geodesic Photogrammetry System (GPS) which uses photogrammetry for detecting electrodes on a Electric Geodesics EEG cap (Electrical Geodesics, Inc. 500 East 4th Ave. Suite 200, Eugene, OR 97401, USA). This system uses multiple cameras, arranged in a geodesic array. It acquires images of the sensors, allowing the reconstruction of their positions in space. Further, it does not require that any device touches the subject or the electrodes. However, this system works exclusively with the EGI's geodesic sensor net and it still takes 15–20 min to mark the sensor positions for a 128 system. The ANT Neuro Xensor (ANT Neuro, Colosseum 22, 7521 PT, Enschede, Netherlands) is a similar system to the FastTrack and ELPOS but in its turn it uses infrared light and reflectors to detect the position of the stylus and calculate the electrodes' positions. The Xensor uses a similar procedure as the ELPOS and FastTrack thus potentially inducing error. Its acquisition time is long and user dependent.

These systems offer a variable accuracy and are often not reliable (Michel et al., 2004; Engels et al., 2010). Also, they work with dedicated systems as the EGI geodesic system or require user intervention. Inaccurate electrode localization has an impact on source activity determination. De Munck et al. (1991) reported a source determination error of 4 mm for a 2.5 mm sensor localization error with a linear relationship between error of source localization and error of electrode localization. Wang and Gotman (2001) too reports that an average of 5 mm sensor localization error results in a source dipole fitting error of 5 mm. These authors further discuss the impact and relationship of noise and sensor localization on source localization accuracy. Signal noise, just as sensor mislocalization, is a major source of error for inaccurate source localization. Even so, we must also address the problems of accurate sensor localization, therefore, more systematic studies of evaluation of present methodology are necessary to point out the methods' problems and strengths in a consistent, reliable form.

Perhaps a more reliable and user independent method for acquiring the electrodes' positions is the one described by the patent EP 2 561 810 A1, WO 2013 026 749 from Engels et al. (2011). This method is semi-automatic, uses flat reflector markers on top of the electrodes and at least 14 cameras arranged around the subject. However, this method also needs a MRI scan of the persons' head and a laser digitized scan of a part of the persons' face and head, which can be somehow impractical.

With this in mind we tried to create a method that is fast, accurate and easy to use as well as user independent. In this article we present a method which employs the use of an Infrared Motion Capture system (IR-MOCAP). We call this method System for Spatial Detection of Electrodes or SSDEL. With this article we only attempt to share with the community how to use an IR-MOCAP system for the digitization of EEG electrodes and how accurate and reliable it can be. Issues such as comparison with other methods, costs, required physical space and others, are not subject of this paper.

## 2. Method description

**Figure 1:**
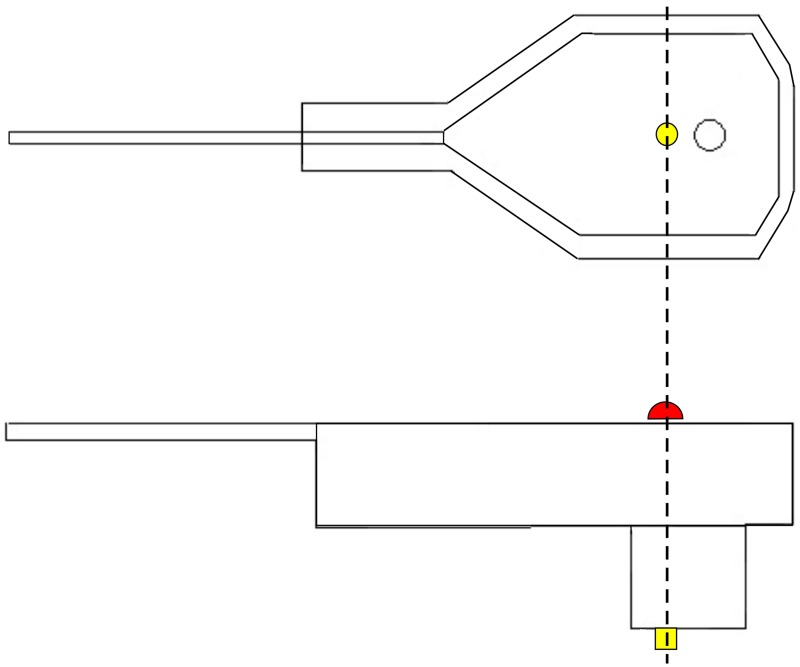
**Graphical representation of an EEG electrode**. On top, a lateral view of the electrode. On the bottom, we have a view from the top surface of the electrode. In yellow, we have the electrode pin, which receives electrical activity. In red, we have the depiction of the reflector marker. As we can see from this figure, the center of the reflector corresponds exactly to the center of the pin.

SSDEL uses 4 mm, or larger, hemispherical passive reflectors on the top surface of each electrode, directly above the electrode pin as illustrated in Figure 1. The reflectors are covered with a retro reflective micro glass beads coating to permit light reflection. Alternatively, active markers can also be used. These reflectors are accurately fixed on the sensor by means of hot glue. This kind of glue does not corrode the reflector or electrode plastic material and is a strong adhesive. Hemispherical reflectors are preferable to flat ones because they increase the area of visibility, i.e., each camera will be able to see more reflectors than if they were flat. Furthermore, the system detects the center of a sphere, and therefore, by calculating the center of the half sphere we will obtain the exact point at the surface of the electrode, corresponding to the pin position. This substantially increases the accuracy of the system.

**Figure 2:**
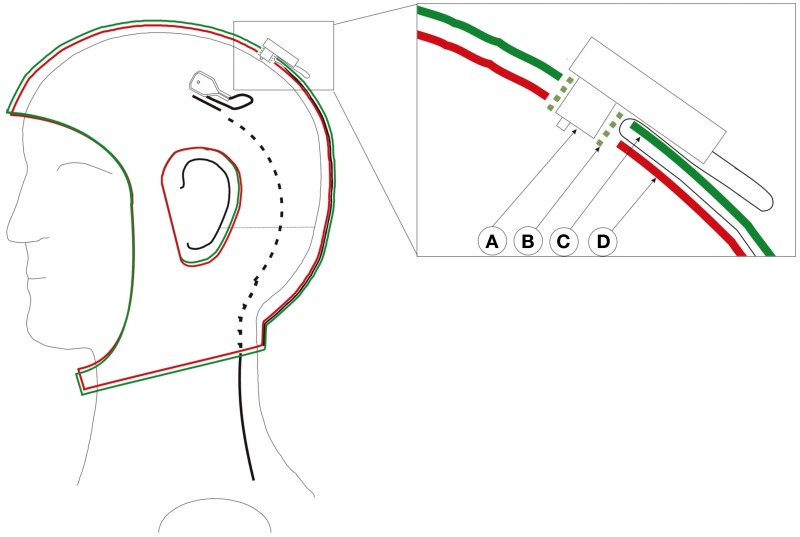
**Custom adapted double-layered actiCap**. This cap holds all cables preventing cable movements and avoid that cables cover the reflector, therefore preventing the interference of the cables on the image capture process. **Left** cap with an electrode whose black wire enters into the cap layer. The black cable turns from a full line to a dashed line as it enters the cap. **Right** close-up of a transverse view of an electrode inserted in the cap. A: Electrode. B: Electrode holder. C: Upper cap layer. D: Lower cap layer. The green plastic electrode holder helps to fix both layers and the electrode. The cable passes through the first layer and stays fixed between both layers. Figure adopted with permission from Reis et al. (2014).

All other parts of the electrodes, which could reflect light, are covered with black matte RAL 9011 plastics paint as to avoid undesirable reflections. Additionally, SSDEL uses a double-layered cap with the electrodes' wires between both layers as seen in Figure 2. This stops the cables from touching the reflectors, therefore halts the interference of the cables on the image capture process. The used cap is described in Reis et al. (2014). This is a modified actiCAP from Brain Products (Brain Products GmbH, 82205 Gilching, Germany).

**Figure 3:**
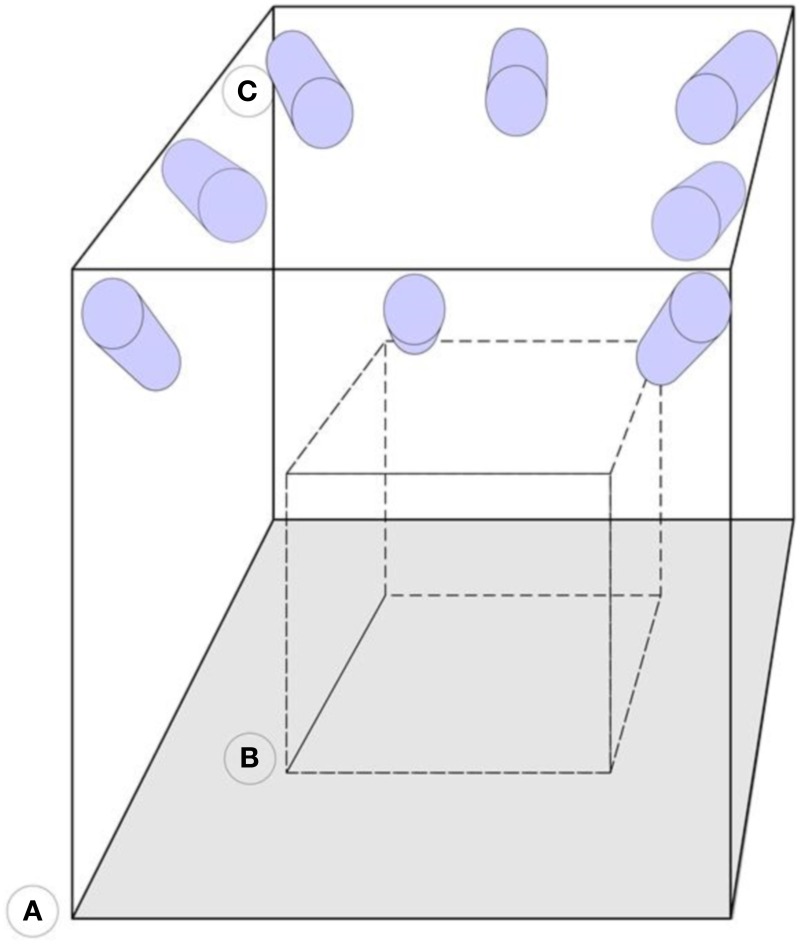
**Representation of the camera arrangement. (A)** Motion capture laboratory. **(B)** Calibrated volume. **(C)** Infrared sensitive cameras. The cameras are mounted on the walls and focused on the same calibrated area volume.

The arrangement of the electrodes is irrelevant as the system works with any pattern of electrode arrangement as long as the reflectors are visible. Further, the system resorts to a stable structure that supports a minimum of six cameras, this can be walls or tripods. Tripods are not advisable because they are movable, but if measurements take place outside the laboratory, then tripods may prove useful. The cameras are infrared sensitive and have an infrared light emission ring around the lenses, which increases the markers visibility. These cameras are about 1 m in average away from and around the focus points, with about 1.5 m distance between each other and are all focused and calibrated to measure the same volume in between them. Inside this volume, the subject, wearing the EEG cap, sits in a way that each reflector is seen by at least three cameras. Figure 3 illustrates the camera setup and measuring volume. Within this volume, the subject is mostly free to move due to the arrangement of the cameras that allow the reflectors to be seen in any position inside the calibrated volume.

In fact, SSDEL can potentially be used to capture electrode positions during motion or physical exercise, such as running on a treadmill or cycling. This is useful to compensate for small changes in electrode positions that occur during head movement. After the cameras are arranged, calibration of the system follows. We calibrated the system as advised by the cameras manufacturer, using the wand calibration method. This method uses a calibration wand and an L shaped reference. These determine the volume and the origin or the measurement volume coordinates and the Xx and Yy axis. The calibration wand is moved inside the measurement volume in all three dimensions. The system then calculates, by triangulation, the relative positions and orientation of each camera by analysing the camera's views of the wand. To localize a marker, it must be seen at least two cameras. This provides an accurate calculation of the XYZ coordinates in the calibrated general coordinate system.

**Figure 4:**
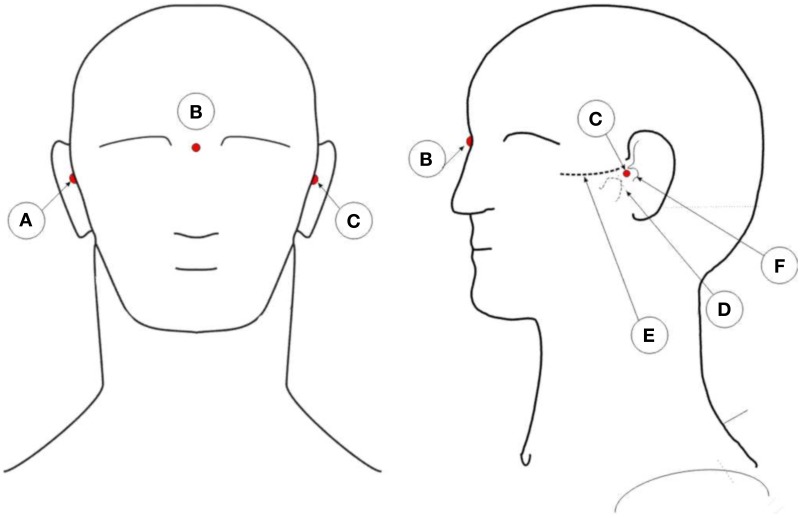
**Illustration of the placement positions of anatomic reference reflectors**. **A**: Right pre-auricular. **B**: Nasion. **C**: Left pre-auricular. **D**: Condyloid process. **E**: Zygomatic arch. **F**: Tragus.

The captured XYZ coordinates of the reflectors need to be co-registered with the person individual anatomy. To co-register the coordinates with the person's head, three additional markers are placed on the subject's head. Figure 4 illustrates the position of these points. One marker on the pre-auricular area between the upper posterior part of condyloid process and the tragus, on each side of the head. This is a point on the posterior root of the zygomatic arch immediately in front of the external acoustic meatus. The third marker finds its place at the nasion position. These three points are used to calculate the center of the head, which is the point of interception between a line connecting both pre-auricular points and a perpendicular line from the resulting line to the nasion marker. With these three points, researchers can calculate the center of the head of the subject. From this point, re-reference all the electrodes to the calculated head center. The electrodes' thickness is then subtracted from the captured XYZ values of each electrode reflector coordinate by subtracting the thickness of the electrode to the total distance of the electrode top to the center of the sphere. All these calculations can be performed in MATLAB (MathWorks, Natick, Massachusetts, United States of America) or other program such as the free software GNU Octave (https://www.gnu.org/software/octave/index.html). From MATLAB, researchers can export the data to other software formats as they see fit.

The described method involves the recording of a set of synchronized images of the reflectors over time. For the measurements we use a sample rate of 100 Hz over 10 s. This results in 1000 data points for each reflector that are used to view the position of the electrodes over time. The user can easily discard, manually, falsely detected or noisy points and select only the time points that contain the 3D coordinates of interest. This system uses Oqus 300+ infrared light cameras with high-speed video capability which permit image capture at full resolution up to 500 frames per second (fps) or reduced resolution up to 10,000 fps. (Qualisys AB, Gothenburg, Sweden), additionally we suggest the use of the Track Manager software also provided by this company for capturing the 3D data points.

Labeling and identification of the detected points involves the manual creation of a first model or template, which thereafter can be automatically fitted into subsequent captures for every person. The user should create this template model by manually labeling each of the detected points. Each time a new set of electrodes is measured this data should be added to the model so that it accounts for differences in the various measurements, therefore increasing the efficiency of the automatic model fitting. The automated identification of the data points is done by measuring distances and angles between the various markers. The software Track Manager offers such a model that can be used for this purpose (Qualisys AB, Gothenburg, Sweden).

## 3. Comparison with established method

To validate and compare the presented method we considered comparing it with the FastTrack system. However due to these system limitations (Engels et al., 2010), we chose to compare the presented method against values obtained with X-ray computer tomography (CT) scan which should provide us with the most approximate values to reality. Furthermore, CT scanners are a very widely established imaging method, thoroughly known and validated.

### 3.1. Methods

**Figure 5:**
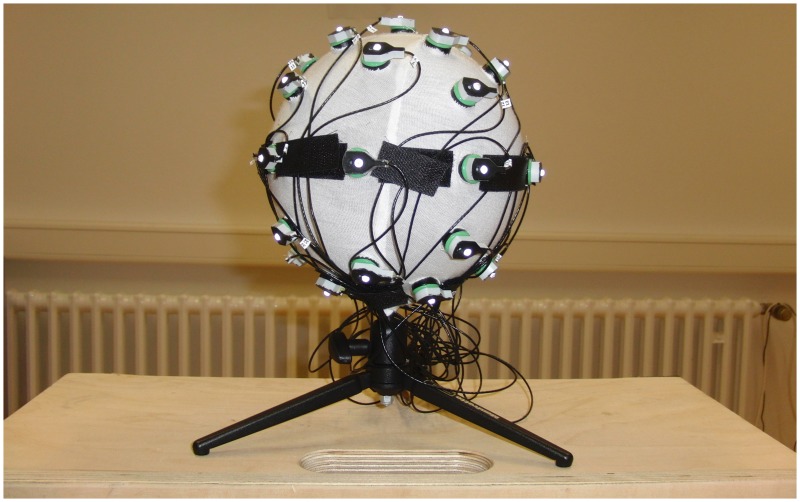
**Photo of the fiberglass sphere**. Here we can see the fiberglass sphere with the attached electrodes, the covering fabric and the hook straps fixing the electrode cables.

We fixed 32 electrodes, with a reflector marker on each, on a precisely manufactured fiberglass sphere with high precision drilled equidistant holes, identical to the ones of the electrode cap. We fixed the electrodes to the sphere in order to avoid changes in electrode positions. The sphere has 16 cm of diameter and stands on a tripod mount. We also covered the surface of the sphere with a non-reflective white fabric and fixed the electrode cables with hook-strap band so to avoid undesirable reflections and cable interference during image acquisition. On Figure 5, we can see a picture of the fiberglass sphere just described above.

**Figure 6:**
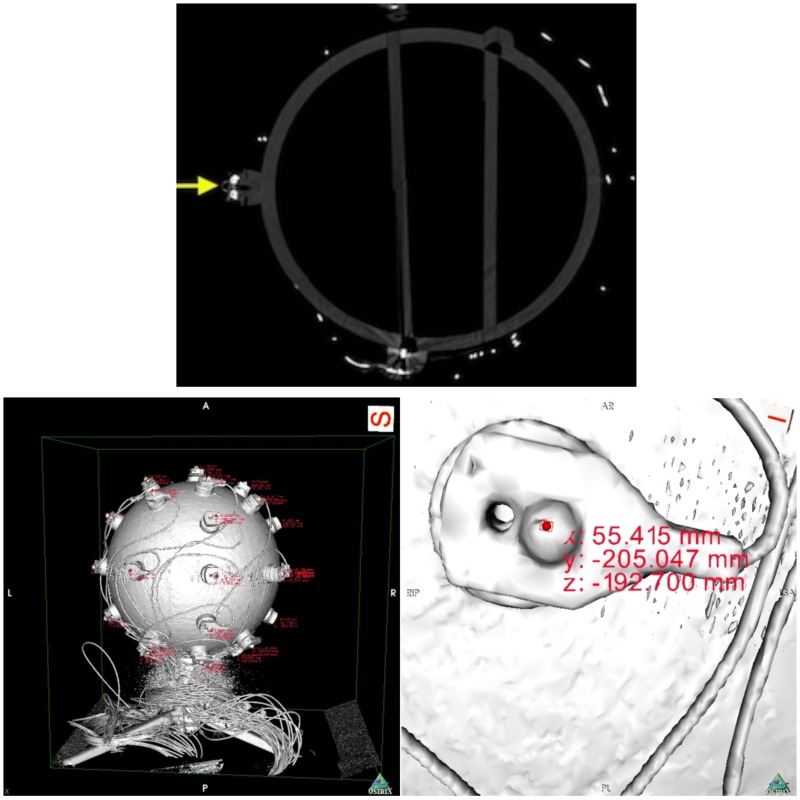
**On top, a slice of the fiberglass sphere CT scan**. The solid yellow arrow points at a sectioned electrode with a reflector on top. On the bottom, images of the sphere's reconstructed surface. On the left, and on the right a close up of an electrode. The pictures shows the reconstructed surface of the sphere and electrodes, obtained from the scan slices. The areas of red text are coordinates that are displayed when an investigator clicks on a part of the digitized model, in this case the top surface of the reflector markers. On the right, we can see the electrode with its reflector and corresponding displayed coordinates.

Next, we proceeded with data collection. We scanned the sphere with a calibrated Siemens Somatom Definition AS X-Ray Computerized Tomograph (Siemens AG, Erlangen, Germany). For the CT scan we used the HeadSpiral 0.6 mm H70h protocol that resulted in 318 slices. This provided a very clean, artifact free imaging of the sphere. The top picture of Figure 6 shows one slice of the sphere resultant from the scan. Afterwards, in a room nearby, we collected data using the SSDEL method with 8 cameras as previously described in Section 2. The system was calibrated by means of wand calibration. Data was collected using the Track Manager software with 100 Hz sample rate and 10 s capture time.

Data analysis followed with the extraction of the coordinates from the CT data. Using the OsiriX open source DICOM viewer software (Rosset et al., 2004). This free software package includes a module for 3D surface reconstruction, thus we reconstructed the sphere model with −300 for pixel value for first surface reconstruction. The results can be observed on the bottom pictures of Figure 6.

With the reconstructed surface model, we were able to extract the coordinates corresponding to each reflector marker. An expert user selected the point corresponding to the top extremity of the reflector by means of a computer mouse. For each click, the expert recorded the value that appeared. According to the law of the classic test theory, reliability can increase by repeating measurements (Lienert and Raatz, 1998). Therefore, we measured each electrode position six times and averaged the results. The criteria and procedure for determining the place of the marker to select was:

1. Zoom in to the electrode as to have the marker on the center of the screen in a view from top perspective;
2. Click on the highest prominence of the marker;
3. No changes to the view position between clicks;
4. Click always on the same most central spot of the marker;
5. Calculation of mean values;
6. Record every coordinate (mean values).

We recorded and calculated the values in a spreadsheet from Microsoft Excel (Microsoft, Microsoft Redmond Campus, Redmond, Washington, U.S.A.).

To the points obtained in 3D by the IR-MOCAP system cameras, we applied a previous constructed model that identified and labeled the markers automatically. Any noisy, or not fully captured, marker was manually deleted. From the TrackManager we exported all data to MATLAB and performed the following calculations. To normalize the data from both systems the points obtained had to be referenced to the sphere model coordinate system. To do this, two points at the equator line of the sphere and opposite to each other were selected (L_90 and R_90) and we calculated a line connecting these two. We selected another point, also at the equator line and at the middle of the two mentioned before (V_90). From this last selected point a line was calculated so that it intercepts the previous calculated line in a perpendicular. The interception point is the calculated center of the sphere. All electrode positions were referenced to this center.

### 3.2. Results from sphere model measurements

The results and obtained coordinates, in millimeters, for each system are presented in the tables below. These values were calculated in Microsoft Excel. All results are relative to the sphere model coordinates' system in which the origin is the calculated center of the sphere. Table 1 shows the Euclidean distances between the CT and the SSDEL obtained points. In Table 2 we can see the average Euclidean distance and standard deviation between both methods. Euclidean distances ranged from 2.31 mm to 0.44 mm.

**Table 1:** **Euclidean distances between each electrode located by the CT and SSDEL**.

**Label**	**Euclidean distances (mm)**
O_90	1.53
LH_45	0.77
RH_45	1.22
RV_45	1.85
LV_45	0.89
LHO_45	0.94
RHO_45	1.75
RVO_45	2.31
LVO_45	0.77
LHU_45	1.57
RHU_45	1.09
RVU_45	0.83
LVU_45	0.44
LO1_30	0.70
HO1_30	1.46
RO1_30	0.68
VO1_30	1.28
LO2_30	0.76
HO2_30	1.20
RO2_30	1.91
VO2_30	1.85
LU1_30	0.79
HU1_30	1.09
RU1_30	0.51
VU1_30	1.75
LU2_30	1.27
HU2_30	1.56
RU2_30	2.28
VU2_30	1.55
H_90	1.27

**Table 2:** **Euclidean distances for each electrode**.

Average distance (mm)	1.26
ST Dev. of distances (mm)	0.51
Max	2.31
Min	0.44

## 4. Measurement with a human participant

In this section, we will see the system capturing the positions of the electrodes while on a person's head.

### 4.1. Data collection and analysis

**Figure 7:**
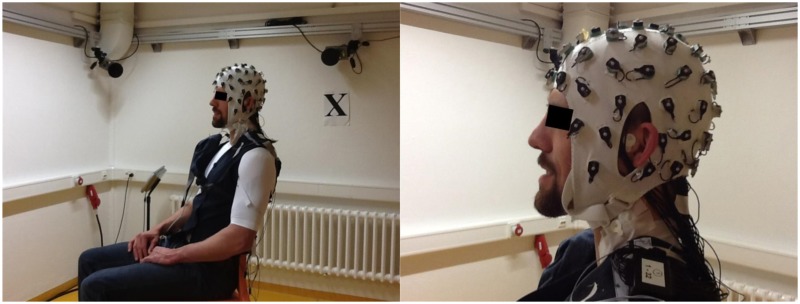
**Image of the Human volunteer sitting during image acquisition wearing a modified 64 Channel actiCAP EEG cap**.

We measured electrodes positions from a volunteer to test this method feasibility in acquiring electrode positions from a human head. Data collection from the volunteer was approved by the ethics committee of the Friedrich-Alexander University Erlangen-Nürnberg. We also obtained written informed consent from the volunteer before conducting this experiment. The system acquired electrodes positions from the human volunteer wearing a modified 64 Channel actiCAP EEG cap (Figure 2), plus reference, ground electrode and three anatomic reference markers. An expert placed the anatomic reference reflective markers on the volunteer's head by accurately palpating the area and identifying the mastoid process and the tragus. During image capturing, we used a sample rate of 100 Hz for 10 s. All points were captured without noise. As the reader can observe on Figure 7 the volunteer was sitting on a chair during the measurement. We can also see one of the anatomic reference markers and that are no free electrode cables interfering with image acquisition. We proceeded to reference the coordinates to the head coordinate system and co-registering the obtained points with the volunteer's individual anatomy by using the reference anatomic points in the procedure explained earlier in Section 2.

### 4.2. Results from human head measurements

**Figure 8:**
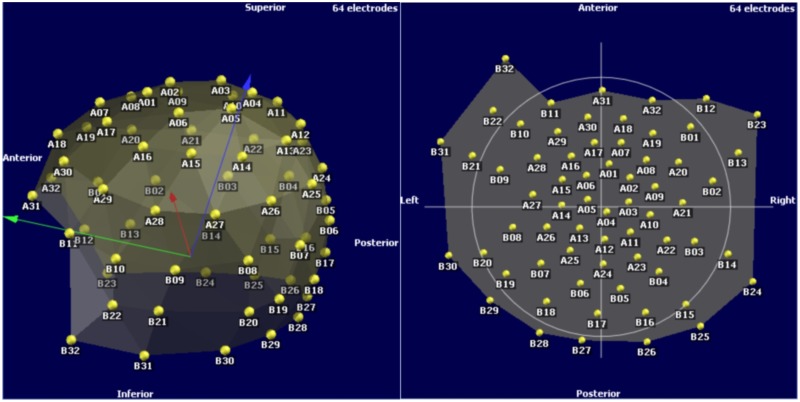
**Electrodes 3D view on a typical EEG analysis software**.

The results are in Figure 8. These are possible to import and utilize with a software that reads custom electrode positions for EEG signal analysis. These images are taken from Cartool (brainmapping.unige.ch/cartool), a free open source software for pre-processing and advanced source localization and analysis of EEG activity.

## 5. Discussion

In this article, we compared the novel SSDEL method against measurements from a CT scanner. We used the results from the CT scanner as the “ground truth” to evaluate the new methodology for determining the 3D electrode positions. The average Euclidean distance between the CT measurements and the new method was 1.26 mm with a standard deviation of 0.51 mm.

In literature, we have not found a coherent way to report the findings so that studies would be comparable. Often authors use creative methodology to validate their new method or simply compare the new method with an established product (Russell et al., 2005; Ettl et al., 2013). This process creates large variability on the pool of information and inconsistencies. Therefore, we choose a known established method that is highly reliable, a CT scan, for validating our method and hopefully new methods that will emerge in the future will do so as well. Nevertheless, the obtained results are comparable with results reported in previous studies: (Russell et al., 2005) reported a mean error of 1.27 mm for the geodesic photogrammetry method and 1.02 mm for the FastTrack method. Here we report for the SSDEL system a deviation ranged from 0.44 mm to 2.31 mm. However, these comparisons are bound to not be much reliable for comparison, due to the factors mentioned above, i.e., no single ground truth measurement method and different study designs. Thus, the main objective of this article is to describe the use of SSDEL for EEG electrodes positioning.

SSDEL is capable of quick, easy and accurate results. The experiment with the fiberglass sphere model shows the accuracy of the system against the measurements of a CT scanner and the second experiment shows that it can easily measure positions on a person's head while he or she wears the EEG cap. Furthermore, this system, since it makes use of a MOCAP system, has the potential to measure electrode positions during movement or exercise, such as treadmill running or cycling. This may prove quite useful as today's behavioral neuroscience is developing toward EEG brain-computer interfaces and recording brain activity during exercise. We can already see the appearance of the first toolbox (Makeig et al., 2009; Ojeda et al., 2014), http://sccn.ucsd.edu/wiki/MoBILAB part of EEGLAB toolbox (Delorme and Makeig, 2004), and mobile EEG systems specialized for mobile brain imaging (Debener et al., 2012; Reis et al., 2014). Presently, at our laboratory, we are working on improving and optimizing the capture of EEG electrodes positions during movement. In the future we will integrate this method with mobile brain imaging techniques.

### 5.1. Limitations of the presented method

The advantage of the SSDEL is in its reliability, subject comfort, and ease of use. Moreover, it is easy to apply in most systems and it automatically identifies the markers. The time it takes to digitize the points is about 10 s, user independent and without touching the sensors.

A limitation is the price of the equipment. SSDEL uses parts of a motion capture system composed of infrared light sensitive cameras and these cameras are more expensive than normal cameras. The system is also subject to undesirable reflections from objects or other reflectors that simply reflect light back at the camera. Moreover, the system occupies a certain space that is necessary for the cameras to have enough viewing angle. The necessary space is normally smaller for other systems, for example the FastTrack system. SSDEL does not record head shape information. The system uses the Track Manager software for recording, locating and identifying the electrodes. For automatic identification, a model template of the positions needs to be constructed by identifying them once, manually.

## 6. Conclusions

In this paper, we presented and described a new method for spatial localization of EEG electrodes. This method, which we call SSDEL, utilizes principles and parts of an IR-MOCAP to acquire the position of markers on top of each EEG electrode. This enables the quick, accurate, user independent, contactless, automatic, and real time detection of each marker. We compared this method with CT measurements of a fiberglass sphere model capable of firmly holding EEG electrodes. We used these measurements as the ground truth for comparison. Then we acquired electrode positions, from the same sphere model, using the new method and determined the Euclidean distances between the measurements of each system, for every electrode. The new method is capable of accurate measurement of the electrode positions, average distance of 1.26 mm and standard deviation of 0.51 mm. These results are comparable with the adopted gold standard CT measurements. Although in our laboratory, we possess two other systems for digitization of electrode positions, this tool has become the main and only used one because of its good accuracy and time economy. It is possible that in the future this tool will become increasingly popular in mobile brain imaging and clinical use due to its accuracy and quick acquisition of data.

## Language corrections

Text revision and English language corrections were done by Andreas Oikonomou, B. A. and Titus Czyz B. A.

### Conflict of interest statement

The authors declare that the research was conducted in the absence of any commercial or financial relationships that could be construed as a potential conflict of interest.
